# CO_2_ Capture from High-Humidity Flue Gas Using a Stable Metal–Organic Framework

**DOI:** 10.3390/molecules27175608

**Published:** 2022-08-31

**Authors:** Qi Wang, Yang Chen, Puxu Liu, Yi Wang, Jiangfeng Yang, Jinping Li, Libo Li

**Affiliations:** Shanxi Key Laboratory of Gas Energy Efficient and Clean Utilization, College of Chemical Engineering and Technology, Taiyuan University of Technology, Taiyuan 030024, China

**Keywords:** CO_2_ capture, ZIF, high humidity, stable structure

## Abstract

The flue gas from fossil fuel power plants is a long-term stable and concentrated emission source of CO_2_, and it is imperative to reduce its emission. Adsorbents have played a pivotal role in reducing CO_2_ emissions in recent years, but the presence of water vapor in flue gas poses a challenge to the stability of adsorbents. In this study, ZIF-94, one of the ZIF adsorbents, showed good CO_2_ uptake (53.30 cm^3^/g), and the calculated CO_2_/N_2_ (15:85, *v*/*v*) selectivity was 54.12 at 298 K. Because of its excellent structural and performance stability under humid conditions, the CO_2_/N_2_ mixture was still well-separated on ZIF-94 with a separation time of 30.4 min when the relative humidity was as high as 99.2%, which was similar to the separation time of the dry gas experiments (33.2 min). These results pointed to the enormous potential applications of ZIF-94 for CO_2_/N_2_ separation under high humidity conditions in industrial settings.

## 1. Introduction

With the rapid growth of economic development needs and the world population, the world’s energy demand is growing by leaps and bounds. Fossil energy will still account for more than 75% of total global energy consumption by 2040 [1]. The massive consumption of fossil energy leads to a continuous increase in the concentration of CO_2_ and other greenhouse gases in the atmosphere, and its excessive emission contributes to the continuous increase in the greenhouse effect [2], which has negative impacts on the global climate. Because CO_2_ is the most important of the greenhouse gases, reducing its emissions is regarded as the most important way to solve the climate problem. Therefore, doing so will also be an unavoidable responsibility and obligation in the next few years.

Among the various sources of CO_2_, emissions of CO_2_ in flue gas from coal-fired power plants account for 30–40% of the total [3]. The main components of flue gas tend to be around 12–15% CO_2_, 85% N_2_, and water vapor [4]. It is most effective and crucial to capture CO_2_ from flue gas for storage due to its large and relatively concentrated CO_2_ emissions. Carbon capture and storage (CCS) is one of the channels to achieving a reduction in CO_2_ emissions. The key factor for significant progress lies in the choice of materials used to perform the separation. In this regard, the main methods for CO_2_ separation are cryogenic fractionation [5], solvent absorption [6], sorbent adsorption [7], bioimmobilization [8], membrane permeation separation [9], and so on. The method of sorbent adsorption has received extensive attention due to its advantages of simple equipment, convenient operation, low energy consumption, and low corrosion and pollution. Zeolite, silica, BPL carbon, and metal–organic framework (MOF) are the most widely used adsorbents by far. In particular, zeolitic imidazolate frameworks [10,11] (ZIF as a subfamily of MOF) have many attractive properties such as high specific surface area, good structural stability, and flexibility that can be modified with functional groups by postsynthetic methods [12,13,14,15]. The framework structure can be rationally designed to achieve a large range of pore sizes and three-dimensional (3-D) pore structures [16,17]. These are also the reasons why ZIF materials can be widely used for sensors [18,19], catalysts [20,21], catalyst supports, selective layers on catalysts via encapsulation [22,23], adsorbents [24,25,26], matrix fillers [27,28,29], membranes [30,31,32,33,34,35,36,37], and drug delivery systems [38,39,40]. ZIF materials play a pivotal role in the adsorption and separation of CO_2_ under dry conditions based on these merits, such as ZIF-68–82 with the gmelinite (zeolite code GME) topology reported by Yaghi et al. [41]. The CO_2_ adsorption capacity of ZIF-78 reached 49.03 cm^3^/g at 760 Torr and 298 K; they also reported two kinds of porous materials [42], ZIF-95 and ZIF-100, for which the adsorption capacities for CO_2_ were 19.26 cm^3^/g and 21.28 cm^3^/g, respectively, under the conditions of 298 K and 1 bar.

Although many materials have impressive CO_2_ capture capabilities in dry environments, it is worth mentioning that the presence of water in flue gas during practical applications, in addition to higher CO_2_ capture capacity, makes it necessary to find novel adsorbents that have higher tolerance for humid environments. A few studies have been conducted on the hydrolytic stability of MOFs in humid environments. MIL-110/-101, CPO-27, and ZIF-8 are stable materials when exposed to water vapor for 2 h [43]. UiO-66-NH_2_ [44] can maintain its original crystallinity and porous structure before and after water adsorption (relative humidity of 80%). MIL-100 [45] is completely water-stable owing to its trinuclear chromium clusters. The surface of MIL-53 is transformed, yielding H_2_BDC and γ-AlO(OH) in boiling water [46]. ZIF-8 and ZIF-94 (also known as SIM-1) are stable after treatment in boiling water for at least 24 h [47,48,49]. Among them, the CO_2_ adsorption capacity under dry conditions of ZIF-8 is 15.64 cm^3^/g at 298 K and 1 bar [50]; ZIF-94 also has the same SOD topology as ZIF-8. The former has a smaller pore size of the sodalite cavity due to the existence of the aldehyde group in the ligand, which may have a stronger force on CO_2_ and further increase its CO_2_ adsorption. However, there have been few reports on ZIF materials for CO_2_/N_2_ separation in humid environments.

Therefore, ZIF-94 was synthesized at room temperature using methanol and tetrahydrofuran as solvents in this paper. First, its stability was explored in humid and harsh chemical environments; the gas adsorption performance was then studied, and finally, the adsorption/separation performance of the material for CO_2_/N_2_ mixtures in dry and high humidity environments was studied in detail, confirming that the presence of water vapor has negligible influence on the CO_2_ enrichment process. The above conclusions confirmed that ZIF-94 was a promising candidate for capturing CO_2_ from high-humidity flue gas by virtue of its satisfactory structural stability and reusability.

## 2. Results and Discussion

### 2.1. Synthesis, Structure, and Characterization

**Figure 1:**
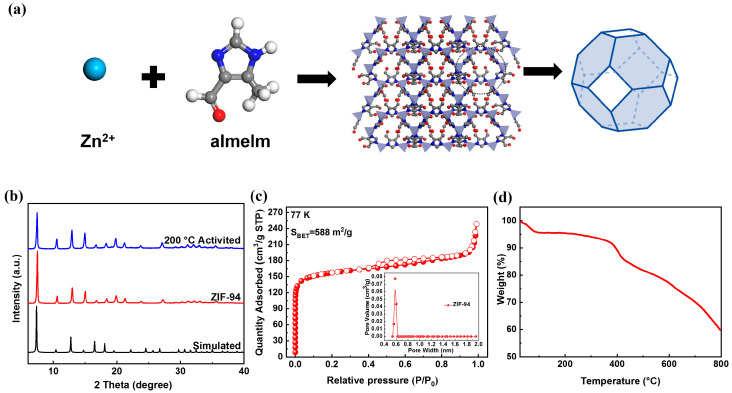
(**a**) The structure of ZIF-94. (**b**) The PXRD of freshly synthesized samples and activated samples. (**c**) The N_2_ adsorption isotherm of ZIF-94 at 77 K. (**d**) TGA data obtained for ZIF-94.

ZIF-94 consisted of Zn tetrahedra linked by carboxylimidazolates forming a 3-D zeolitic imidazolate framework crystallizing in a sodalite topology [51,52]—see Figure 1a. ZIF-94 was built by combining experimental and computational methods, and it is isostructural to ZIF-8 (SOD), which is commercialized under the name Basolite Z-1200™ [12]. ZIF-8 has a methyl group at position 2, whereas ZIF-94 contains a methyl and an aldehyde in positions 4 and 5, respectively. The simulation indicated a pore size of the sodalite cavity of 8.0 Å for ZIF-94 against 11.3 Å for ZIF-8, and the difference in the imidazole group resulted in a smaller cavity for the former [53,54]. As shown in Figure S1, the ZIF-94 was synthesized at room temperature using methanol and tetrahydrofuran, and 3.67 g of product could be synthesized at one time; it has the potential for large-scale synthesis. The freshly synthesized sample appeared as a white crystalline powder, and its powder X-ray diffraction (PXRD) is shown in Figure 1b. The diffraction peaks of the synthesized and activated samples were consistent with the simulation peak of ZIF-94, and the peak shape was relatively sharp, indicating that the sample under this condition had high crystallinity and purity. Scanning electron microscopy (SEM) revealed the morphology and size of ZIF-94. Figure S2 shows that the ZIF-94 crystal had a 3-D structure of a dodecahedron with a smooth surface and a particle size of about 4 nm. The N_2_ adsorption isotherm at 77 K showed the microporous structure of the material (Figure 1c) with a BET surface area of 588 m^2^/g, which was comparable to the reported value [55]. The thermogravimetric (TG) curve of the material in Figure 1d showed the two weight loss steps in the decomposition process. The first may be attributed to the loss of the solvents CH_3_OH and H_2_O in the pores of ZIF-94, and the second may be due to the disintegration of the structure. However, the sample was thermally stable below 400 °C. ZIF-94 synthesized at room temperature with a stable structure offers the possibility for the adsorption and separation of CO_2_ under actual industrial conditions.

### 2.2. Gas Adsorption Performance

**Figure 2:**
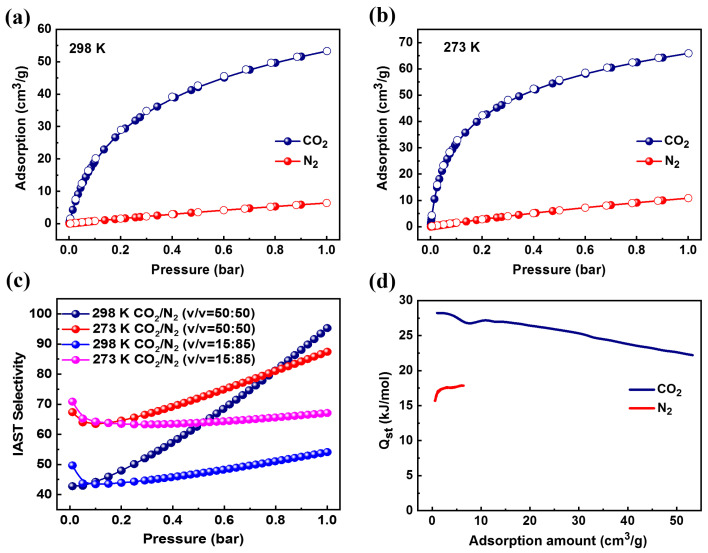
(**a**) The adsorption isotherms of CO_2_ and N_2_ at 298 K and 1 bar. (**b**) The adsorption isotherms of CO_2_ and N_2_ at 273 K and 1 bar. (**c**) The calculated CO_2_/N_2_ selectivity at 273 and 298 K. (**d**) The isosteric heats of adsorption (*Q*_st_) for ZIF-94.

Based on the pore structure and configuration of ZIF-94, the adsorption isotherms of CO_2_/N_2_ were studied at different temperatures (273 and 298 K) and 1 bar. Figure 2a shows the adsorption isotherms of CO_2_ and N_2_ at 298 K and 1 bar. ZIF-94 showed high CO_2_ uptake (53.30 cm^3^/g) and low N_2_ uptake (6.39 cm^3^/g), giving an excellent uptake ratio of CO_2_ over N_2_ (8.34), indicating that the structure had a unique adsorption affinity for CO_2_. An adsorption test was performed at 273 K under the same conditions to study the adsorption performance of the material further, as presented in Figure 2b. Notably, lower temperatures led to higher adsorption capacity for the same component, and for N_2_ under the same conditions, the adsorption capacity of CO_2_ was still higher than that of the former. In addition, the adsorption and desorption isotherms of CO_2_ and N_2_ at the two temperatures overlapped completely, and it could be clearly seen that the adsorption–desorption process of the material could be accomplished perfectly, which could reduce energy consumption and provide the possibility of implementing the adsorption–desorption cycle of the adsorbent in industry. Subsequently, ideal adsorbed solution theory (IAST) calculations were performed using the fitted parameters of the two-site Langmuir–Freundlich isotherm model (Tables S1 and S2) to predict the selectivity of the CO_2_/N_2_ mixture at different temperatures (273 and 298 K) and 1 bar. As shown in Figure 2c, the calculated CO_2_/N_2_ (15:85, *v*/*v*) selectivities were 67.11 and 54.12 at 273 and 298 K, and the calculated CO_2_/N_2_ (50:50, *v*/*v*) selectivities were 87.44 and 95.29 at 273 and 298 K, respectively. The Langmuir–Freundlich parameters are shown in Figure S3 and gave satisfactory agreement (*R*^2^ > 0.999). Therefore, the CO_2_-selective adsorbents were compared in terms of CO_2_ adsorption capacity and selectivity, as shown in Table S3, outperforming previously reported benchmark materials with GME topology (such as ZIF-78 and ZIF-82), which showed the great potential of ZIF-94 in CO_2_/N_2_ separation. In addition, the heats of adsorption of CO_2_ and N_2_ of ZIF-94 as shown in Figure 2d were calculated using the Clausius–Clapeyron equation. In the near-zero adsorption region, the adsorption heats of ZIF-94 for CO_2_ and N_2_ were 28.23 kJ/mol and 15.71 kJ/mol, respectively. On the one hand, this showed that ZIF-94 was a CO_2_-selective adsorbent, and on the other hand, the relatively low adsorption heat was beneficial to the desorption regeneration of the adsorbent during the separation process, and the view was also confirmed that the above adsorption–desorption isotherms were completely coincident (Figure 2a,b), further reducing the energy consumption. In addition, ZIF-94 showed high adsorption capacity and high IAST selectivity for CO_2_/N_2_ mixtures, making it an excellent potential adsorbent for high-humidity applications.

### 2.3. Dynamic Breakthrough Experiments for CO_2_/N_2_ Mixtures

**Figure 3:**
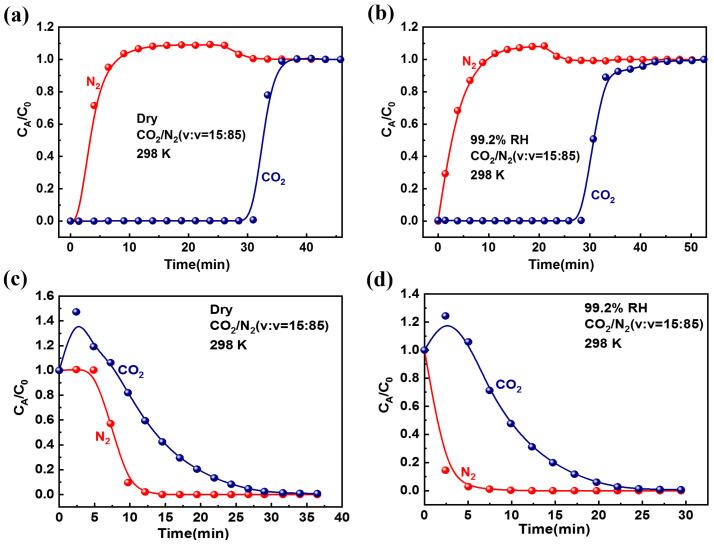
Experimental breakthrough and desorption curves for CO_2_/N_2_ mixture (15/85, *v*/*v*) under two humidity conditions: (**a**,**c**) dry gas and (**b**,**d**) wet gas (99.2% RH).

The previous experiments demonstrated that ZIF-94 has high CO_2_ adsorption capacity and satisfactory CO_2_/N_2_ selectivity; the phenomena prompted us to investigate the feasibility of using ZIF-94 in the separation process under humid conditions. Two types of wet breakthrough experiments for CO_2_/N_2_ (15/85; *v*/*v*) were conducted in this section (Figure S4), dry gas and wet gas (99.2%), to evaluate the gas-separation performance under ambient conditions. The feed gas of CO_2_/N_2_ was passed through the adsorption column loaded with activated ZIF powder at a total flow rate of 5 mL/min at 298 K and 1 bar. For the dry gas experiments, as shown in Figure 3a, N_2_ was initially eluted through the adsorption column at 1.4 min and then reached saturation, whereas CO_2_ was selectively adsorbed on the column at both compositions until 33.2 min. At the same time, the CO_2_/N_2_ mixture could be efficiently separated to obtain high-purity CO_2_ within these time intervals. In addition, the gas adsorption capacity of CO_2_ on ZIF-94 reached 32.76 cm^3^/g in dynamic breakthrough experiment. This indicated that during the dynamic adsorption process of the mixed gas, ZIF-94 displayed a stronger adsorption affinity for CO_2_ than for N_2_. Afterward, desorption experiments on CO_2_/N_2_ mixtures purging the column with Ar at a flow rate of 10 mL/min were conducted under ambient conditions. As shown in Figure 3c, the concentration of N_2_ dropped sharply to achieve N_2_ desorption in preference to CO_2_ during the purging time of 9.7 min. Similarly, we could obtain purified CO_2_ at the outlet of the adsorption column until purging for 34.0 min. ZIF-94 achieved desorption of CO_2_ by room-temperature purging in a short time, which provides the possibility of its repeated use in industry.

As a major source of CO_2_, flue gas is particularly important with regard to CO_2_ capture. However, the presence of water in flue gas imposes higher requirements for the CO_2_ adsorbent; it is still necessary to maintain its CO_2_ capture capacity under wet conditions. To make the ZIF-94 more suitable for practical application, a mixture of CO_2_/N_2_/H_2_O(v) under aqueous conditions (RH = 99.2%) was studied, and the rest of the conditions were consistent with the above dry experiments. For the wet gas (99.2%) experiments, as shown in Figure 3b, N_2_ and CO_2_ were first detected through the adsorption column at 1.4 min and 30.4 min, respectively. The dynamic saturated adsorption amounts of CO_2_ and N_2_ are 30.94 and 5.74 cm^3^/g, respectively, and the dynamic selectivity is 30.54. We were delighted to discover that the CO_2_/N_2_ mixture was still well-separated on ZIF-94 when the relative humidity was 99.2%, and the separation time (30.4 min) was similar to the separation time of the dry gas experiments (33.2 min). A possible cause of this phenomenon was that the presence of –CH_3_, a hydrophobic group, makes ZIF-94 insensitive to water molecules. At the same time, it showed its poor adsorption capacity for water vapor at room temperature and pressure (Figure S5). It is worth mentioning that the breakthrough curve in a humid environment showed a longer tail, revealing that the adsorption dynamics were also impacted by water, which was attributed to the increased dependence on molecular mass transfer caused by saturation of the outer adsorbent layer [56]. Figure 3d shows CO_2_ at the outlet of the adsorption column until purging at 29.4 min in the desorption experiments, a slightly shorter time than in the dry gas experiments (Figure 3c). In summary, the two types of wet breakthrough experiments showed similar characteristics regardless of the humidity, and it was inferred that water vapor adsorption had no effect on the separation of CO_2_/N_2_; ZIF-94 had excellent water resistance while maintaining its gas separation performance capture capability in wet conditions.

### 2.4. Structural and Performance Stability Investigations

**Figure 4:**
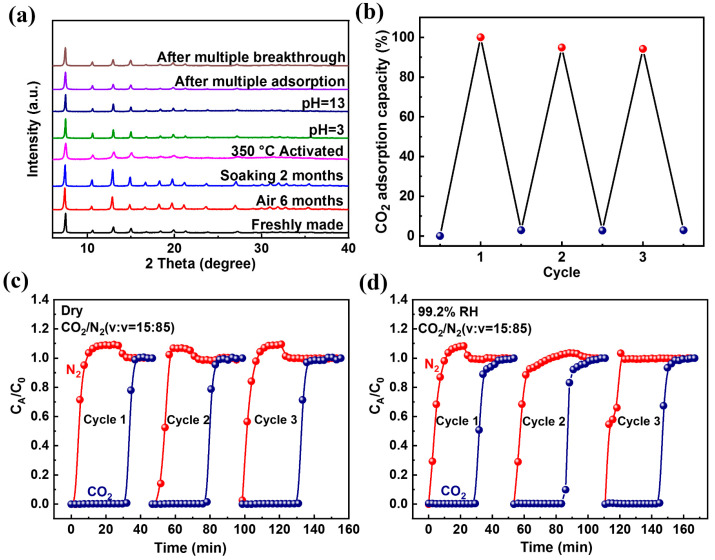
(**a**) Corresponding PXRD patterns to show the maintained structure of ZIF-94 under different conditions. (**b**) Cycling adsorption isotherms for CO_2_ at 298 K and 1 bar. Cycling breakthrough experiments on CO_2_/N_2_ mixture (15/85, *v*/*v*) under two humidity conditions: (**c**) dry gas and (**d**) wet gas (99.2% RH).

In addition to the potential for large-scale synthesis of ZIF-94 at room temperature, we also needed to evaluate it in terms of structural stability and performance stability, so that it can be better used in the separation of CO_2_ in flue gas. In the structural stability assessment, as shown in Figure 4a, the PXRD pattern obtained after being exposed to air for half a year (Figure S6) was still in good agreement with the freshly synthesized sample and had no impurity peaks, indicating that the sample has excellent stability in air. Destruction of the sample structure is often caused by the presence of water molecules, so the stability test of ZIF-94 was conducted in a humid environment. The samples were immersed in water for 1 day, 3 days, 7 days, 1 month, and 2 months (Figure S7); the test results showed that the crystallinity of the sample basically did not change with the prolongation of soaking time. The PXRD patterns of the sample were analyzed after activation at different temperatures (Figure S8), and the PXRD patterns under these conditions are still consistent with the simulated peaks. Moreover, the chemical stability of ZIF-94 was evaluated by treating the samples under harsh conditions. Their PXRD patterns also remained intact after soaking in different pH solutions for 2 days (Figures S9 and S10), and there was no phase transition or structural collapse, demonstrating the material’s resistance to harsh chemical environments. The PXRD patterns of the samples after multiple adsorptions and dynamic breakthrough experiments were compared with those of the freshly synthesized samples, and the intensity of the diffraction peaks was still well-maintained, it can be seen that the above two processes could not destroy the crystal structure. In short, ZIF-94 exhibited satisfactory structural stability, excellent thermal stability, and exceptional recoverability in water and aqueous alkaline solutions. At the same time, ZIF-94 also preliminarily showed that it was a promising candidate for adsorption and separation under high humidity conditions by virtue of its water resistance.

In the performance stability assessment, first, we performed single-component adsorption cycle experiments of CO_2_ at 298 K and 1 bar. As shown in Figure 4b, the adsorption capacity of CO_2_ hardly decreased in adsorption cycling experiments, indicating its good structural stability and reusability. Furthermore, component adsorption of CO_2_ was performed on ZIF-94 under different conditions, as shown in Figure S11. It was found that the CO_2_ adsorption amount of the sample exposed to the air for 4 months was 49.59 cm^3^/g, and the sample soaked in water for 2 months at room temperature (56.33 cm^3^/g) was close to that of the freshly made sample (53.30 cm^3^/g), which further confirms that ZIF-94 maintains its original adsorption capacity in a high-humidity environment. Finally, Figure 4c,d displays cyclic breakthrough experiments using a CO_2_/N_2_ mixture (15/85, *v*/*v*) under the two humidity conditions three times, and the breakthrough time was almost constant, which confirms that the presence of water vapor has negligible influence on the CO_2_ enrichment process. The performed N_2_ adsorption–desorption experiments proved that the N_2_ adsorption amount of the material remained unchanged (Figure S12), and it maintained its initial porous structure. Moreover, ZIF-94 could repeatedly achieve separation of CO_2_/N_2_ without loss of performance in both dry and humid environments. The above test results indicated the excellent cycle performance of ZIF-94, coupled with its robust structural stability. The excellent cycle performance for CO_2_ adsorption and separation points to the enormous potential applications of ZIF-94 for CO_2_/N_2_ separation under humid conditions in industrial settings.

## 3. Materials and Methods

### 3.1. Preparation of ZIF-94

The synthetic methods described in the literature [55,57,58,59,60] were modified as follows:

ZIF-94: 2.64 g of Zn(CH_3_COO)_2_·2H_2_O (12 mmol) was dissolved in 20 mL methanol, and 2.64 g of 4-methyl-5-imidazolecarboxaldehyde (48 mmol) was dissolved in 50 mL tetrahydrofuran. The methanol solution was added to the tetrahydrofuran solution under vigorous stirring. Afterward, the mixture was continuously stirred at room temperature for 16 h. The pale yellow precipitate was filtered and washed with methanol three times. The resulting sample was dried at room temperature in air for 48 h.

### 3.2. Characterization

The crystal structure and crystallinity of the sample were measured using PXRD on a Bruker D8 ADVANCE X-ray diffractometer with a Cu Kα radiation source (λ = 1.5418 Å). Scanning was performed over a 2θ range of 5–40° at a scanning rate of 4°/min. Thermogravimetric analysis (TGA) of the sample was carried out on a thermal analyzer (NETZSCH STA 449F5). The sample was heated at a rate of 10 °C/min in an N_2_ atmosphere. The morphology of the as-synthesized sample was observed and confirmed using SEM on a Hitachi SU8010 instrument. N_2_ adsorption–desorption isotherms were recorded at 77 K on Micromeritics ASAP 2460 and 3020 instruments. Water-adsorption isotherms at 298 K were measured using an automated volumetric adsorption apparatus (Quantachrome). Before measurement, the sample was degassed at 200 °C for 12 h until the residual pressure was below 1 × 10^−6^ Torr and all gases were of 99.999% purity.

### 3.3. Adsorption and Breakthrough Experiments

Single-component gas adsorption isotherms for CO_2_ and N_2_ were recorded on a Micromeritics ASAP 2020 instrument at 273 K and 298 K. The sample was activated at 200 °C under a high vacuum (10^−6^ Torr) for 12 h until no weight loss was observed. High purity CO_2_ (99.999%) and N_2_ (99.999%) were used in the gas adsorption experiments. Before the breakthrough experiments, the sample was degassed and activated at 200 °C for 12 h, and then cooled to room temperature. Approximately 0.7147 g of each sample was loaded into a stainless-steel adsorption column with an inner diameter of 4.57 mm and a length of 120 mm. High purity Ar was used to purge the column at a flow rate of 10 mL/min to remove any residual gas in the pipeline. The adsorption columns loaded with each sample were fixed in an in-house-built device. The flow rate of CO_2_ and N_2_ was adjusted using a pressure valve and flowmeter located at the inlet of the adsorption column. The concentration of CO_2_ and N_2_ was monitored in real time using gas chromatography (490 Micro GC, Palo Alto, CA, USA) at the outlet of the adsorption column. Each experiment was carried out at 298 K, and the flow rate of the CO_2_/N_2_ mixture (15:85, *v*/*v*) was 5 mL/min until saturation was reached (Figure S4). Subsequently, the adsorption bed was regenerated using Ar (10 mL/min). In addition, after the adsorption and breakthrough experiments, the sample was removed, weighed, and analyzed using PXRD.

### 3.4. Ideal Adsorbed Solution Theory Calculations

The single-component adsorption isotherms of CO_2_ and N_2_ on ZIF-94 obtained at 273 K and 298 K were fitted using the dual site Langmuir–Freundlich model proposed by Myers [61] et al.:

$$q=q_{A,sat}\frac{b_{A}p^{\nu A}}{1+b_{A}p^{\nu A}}+q_{B,sat}\frac{b_{B}p^{\nu B}}{1+b_{B}p^{\nu B}}$$

where *q* represents the adsorbed capacity per mass of adsorbent (mol/kg), *q*_A,sat_ and *q*_B,sat_ are the saturation uptake capacities at sites A and B, respectively, *b*_A_ and *b*_B_ represent the constant at adsorption sites A and B, respectively (Figure S3), *P* represents the total pressure of the gas at equilibrium (kPa), and *ν* represents the Freundlich exponent.

The adsorption selectivity is defined as follows:

$$S_{ads}=\frac{q_{A}/q_{B}}{y_{A}/y_{B}}$$

where *q*_A_ and *q*_B_ represent the component molar loadings within the MOF, and *y*_A_ and *y*_B_ are the corresponding mole fractions used in the feed gas mixture.

The parameters are provided in Tables S1 and S2.

## 4. Conclusions

In this work, ZIF-94 was synthesized at room temperature using methanol and tetrahydrofuran as solvents. ZIF-94 exhibited satisfactory structural stability, excellent thermal stability, and exceptional recoverability in water and aqueous alkaline solutions. The CO_2_ uptake and low N_2_ uptake at 298 K and 1 bar were 53.30 cm^3^/g and 6.39 cm^3^/g, respectively. The calculated CO_2_/N_2_ (15:85, *v*/*v*) selectivities were 67.11 and 54.12 at 273 and 298 K, respectively. The separation time (30.4 min) for the wet gas (99.2%) experiments was similar to the separation time for the dry gas experiments (33.2 min) in dynamic breakthrough experiments for CO_2_/N_2_ mixtures. The CO_2_ adsorption amount of the sample soaked in water for 2 months at room temperature (56.33 cm^3^/g) was close to that of the freshly made sample (53.30 cm^3^/g). The presence of water molecules did not affect the adsorption and separation processes of CO_2_ on ZIF-94. ZIF-94 has been shown to be a kind of adsorbent that has the requisite hydrolytic stability for applications in high-humidity environments.

## Data Availability

The data presented in this study are available on request from the corresponding author.

## Supplementary Materials

The following supporting information can be downloaded at: https://www.mdpi.com/article/10.3390/molecules27175608/s1, Figure S1: Schematic representation of synthesis of ZIF-94 at room temperature; Figure S2: SEM of as-synthesized ZIF-94; Figure S3: CO_2_ (a, 298 K, and b, 273 K) and N_2_ (c, 298 K, and d, 273 K) adsorption isotherms in ZIF-94 with dual-site Langmuir–Freundlich model fits; Figure S4: Diagram of breakthrough experimental apparatus; Figure S5: H_2_O adsorption of ZIF-94 at 298 K; Figure S6: PXRD of ZIF-94 exposed to air for 4 months and 6 months; Figure S7: PXRD of ZIF-94 soaked in water for 1, 3, 7, 30, and 60 days; Figure S8: PXRD of ZIF-94 activated at different temperatures; Figure S9: Images of ZIF-94 before and after soaking in different pH solutions. (a) before soaking, (b) after soaking for 2 days; Figure S10: PXRD of ZIF-94 soaked in solutions with different PH values; Figure S11: The adsorption isotherms of CO_2_ under different conditions; Figure S12: The N_2_ adsorption isotherm at 77 K of the samples. (a) Freshly made, (b) Multiple adsorption experiments, (c) Dynamic breakthrough experiments; Table S1: Dual-Langmuir–Freundlich fitting parameters for CO_2_ and N_2_ in ZIF-94 at 298 K; Table S2: Dual-Langmuir–Freundlich fitting parameters for CO_2_ and N_2_ in ZIF-94 at 273 K; Table S3: Comparation of CO_2_ adsorption uptakes and selectivity for CO_2_/N_2_ around the top-performing ZIF; Table S4: Some typical materials with high CO_2_ adsorption capacity. References [13,62,63,64,65,66,67,68,69,70,71,72,73,74,75,76,77,78,79,80] are cited in supplementary materials.

## Sample Availability

Samples of the compounds are not available from the authors.
