# Supplementary material for: CO2 Capture from High-Humidity Flue Gas Using a Stable Metal–Organic Framework
Source: Molecules. 2022 Aug 31;27(17):5608. doi: 10.3390/molecules27175608 (PMC9458099; doi:10.3390/molecules27175608)
Supplement: Supplementary file 1 [file molecules-27-05608-s001.zip › molecules-1873722-supplementary.pdf]

molecules

Supplementary Materials

CO_2 Capture from High Humidity Flue Gas Using a Stable metal–organic framework

Qi Wang [1], Yang Chen [1,*], Puxu Liu [1], Yi Wang [1], Jiangfeng Yang [1], Jinping Li [1] and Libo Li [1,*]

[1] College of Chemical Engineering and Technology, Shanxi Key Laboratory of Gas Energy Efficient and Clean Utilization, Taiyuan University of Technology, Taiyuan 030024, China
* Correspondence: chenyang@tyut.edu.cn (Y.C.); lilibo@tyut.edu.cn (L.L.)

Materials

All chemical and reagents were purchased from commercial suppliers and used without any further purification. Zinc acetate dihydrate ($Zn(CH_3COO)_2{\cdot}2H_2O$, 99%) and tetrahydrofuran(C_4H_8O, 99.9%) were purchased from Innochem. 4-methyl-5-imidazolecarboxaldehyde($C_5H_6N_2O$, 98%) was purchased from Ark. Methanol (AR) and distilled water were purchased from Sinopharm Chemical Reagent Co. Ltd. CO_2 (99.999%), N_2 (99.999%) and mixed gases of CO_2/N_2=15/85 (v/v) were purchased from Beijing Special Gas Co. LTD (China).

Stability experiment

(1) In order to evaluate the water stability of ZIF-94 under humid condition, about 1.5 g of the ZIF-94 is soaked in a vial with 10 mL of deionized water at room temperature. The soaking time are 1 d, 3 d, 7 d, 30 d, and 60 d, respectively.
(2) The samples were soaked in solutions with different PH values to evaluate the acid-base stability, we prepared the solution with pH = 1 ~ 13 used NaOH and HCl aqueous solution at room temperature, and put 0.2 g samples into vials respectively soaking for 48 h.
Finally, the soaked samples were filtered and dried for PXRD analysis, and the stability of the samples in water and aqueous alkaline solutions were observed.

Isosteric heat of adsorption

The isosteric heats of adsorption (Q_{st}) were calculated from the isotherms measured at 273 and 298 K for CO_2 and N_2, respectively. The isotherms were fitted using equation (1).

$$\ln P = \ln N + \frac{1}{T}\sum_{i=0}^{m} a_i N^i + \sum_{i=0}^{n} b_i N^i \quad (1)$$

Where N is the amount of gas adsorbed at pressure P, a and b are virial coefficients and m and n are the numbers of coefficients required to adequately describe the isotherm. Using the fitting parameters obtained from the above equation, Q_{st} could be calculated using equation (2).

$$Q_{st} = -R\sum_{i=0}^{m} a_i N^i \quad (2)$$

Where R is the universal gas constant.

Dynamic saturated adsorption amount of each component ($q_{i,m}$)

To actually determine the adsorption amount at the mixed gas condition, the dynamic saturated adsorption amount of each component ($q_{i,m}$) was calculated based on the breakthrough curves by the equation (3) described as follows:

$$q_{i,m} = \frac{\int_0^{t_0} (F_i\text{-}F_e)\Delta t\text{-}V_{dead}}{m} \quad (3)$$

Where the F_i is the flow rate of specific gas at the inlet of the adsorption column with the unit of cm^3/min, while the F_e represents the effluent flow rate of the corresponding gas species; V_{dead} is the dead volume of the system (cm^3); And m represents the mass of the adsorbent loaded into the adsorption column (g); t_0 is the retention time interval for the gas mixture.

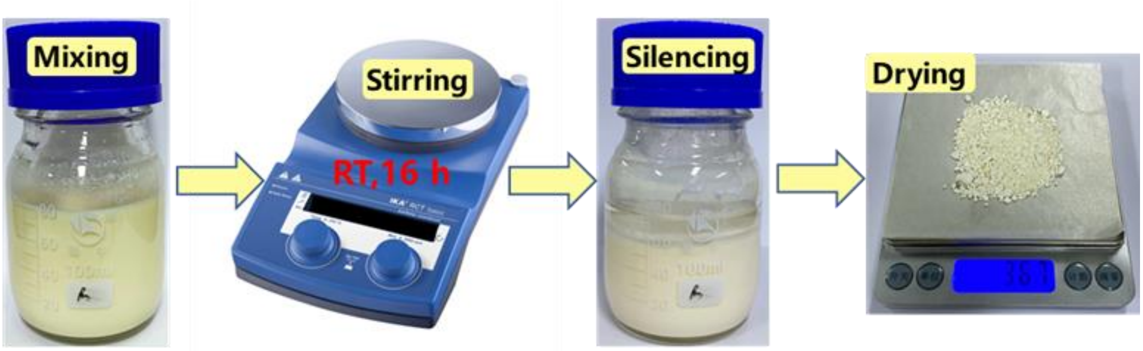

Figure S1. Schematic representation of synthesis of ZIF-94 at room temperature.

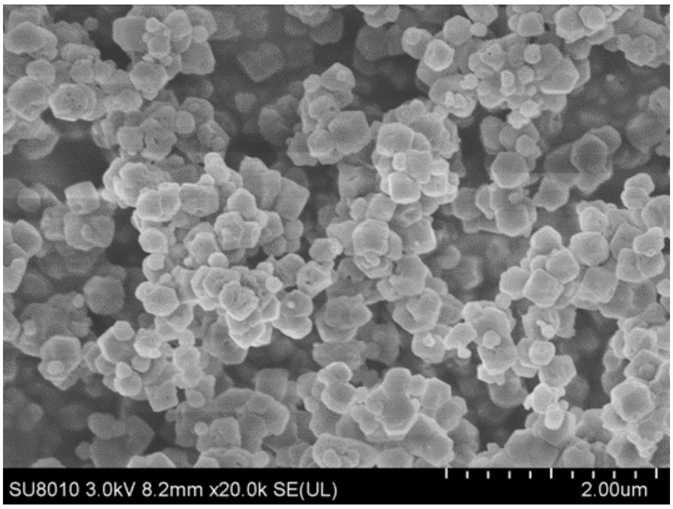

Figure S2. SEM of as-synthesized ZIF-94.

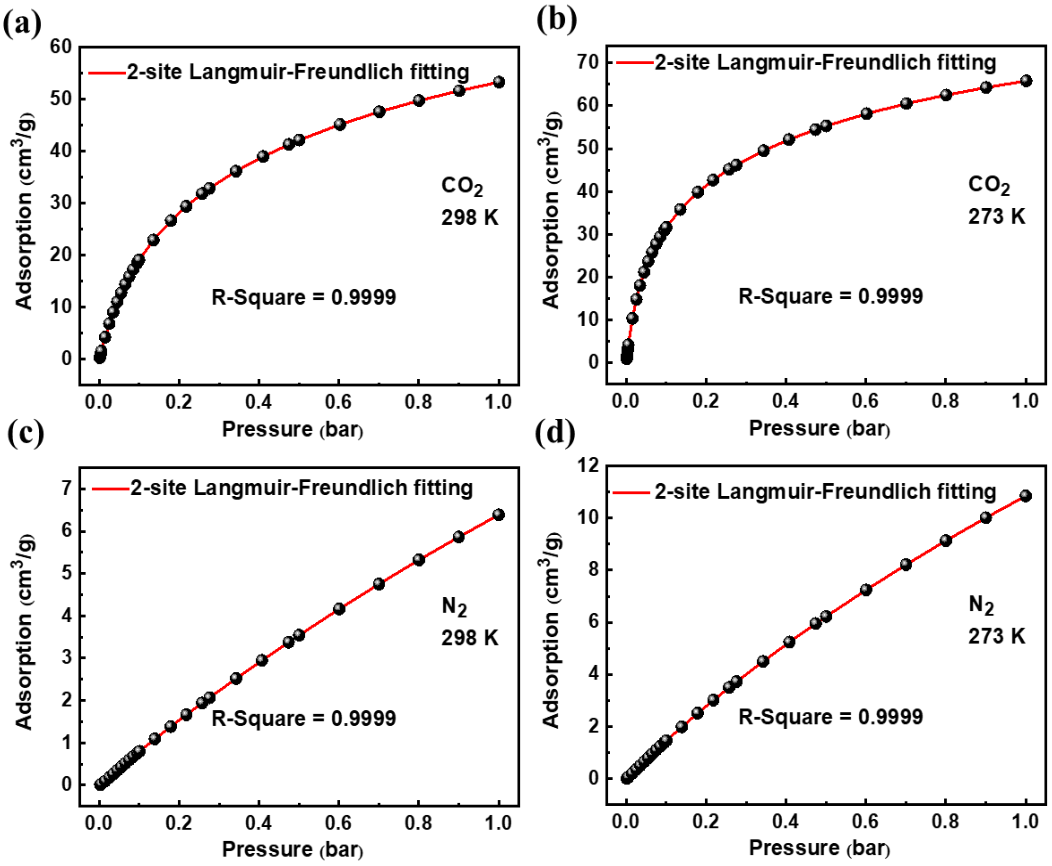

Figure S3. CO_2 (**a**, 298 K and **b**, 273 K) and N_2 (**c**, 298 K and **d**, 273 K) adsorption isotherms in ZIF-94 with dual-site Langmuir-Freundlich model fits.

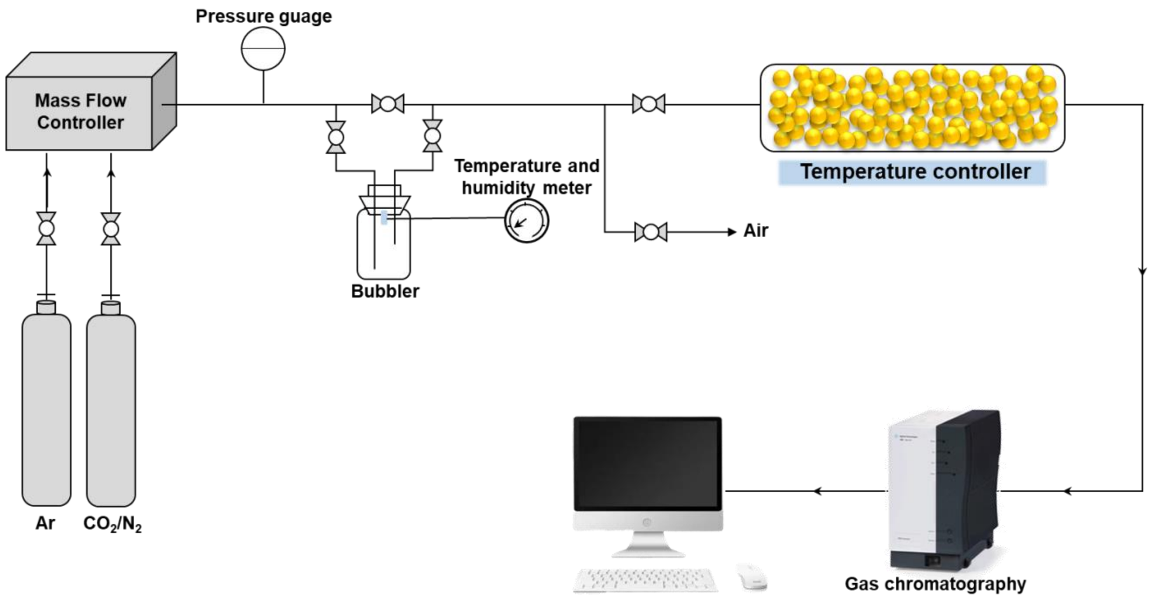

Figure S4. Diagram of breakthrough experimental apparatus.

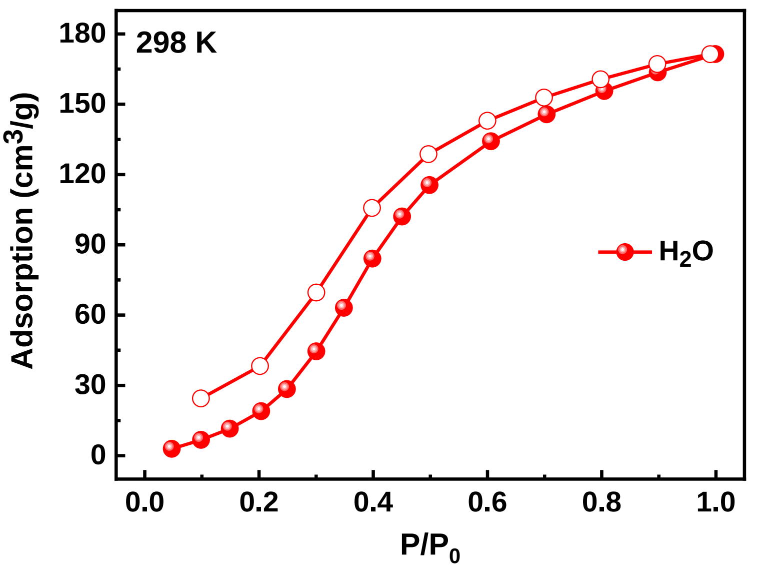

Figure S5. H_2O adsorption of ZIF-94 at 298 K.

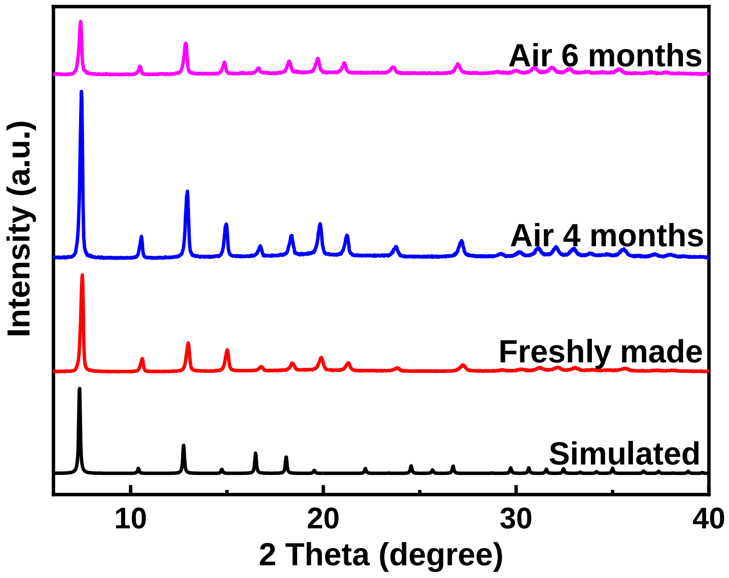

Figure S6. PXRD of ZIF-94 exposed to air for 4 months and 6 months.

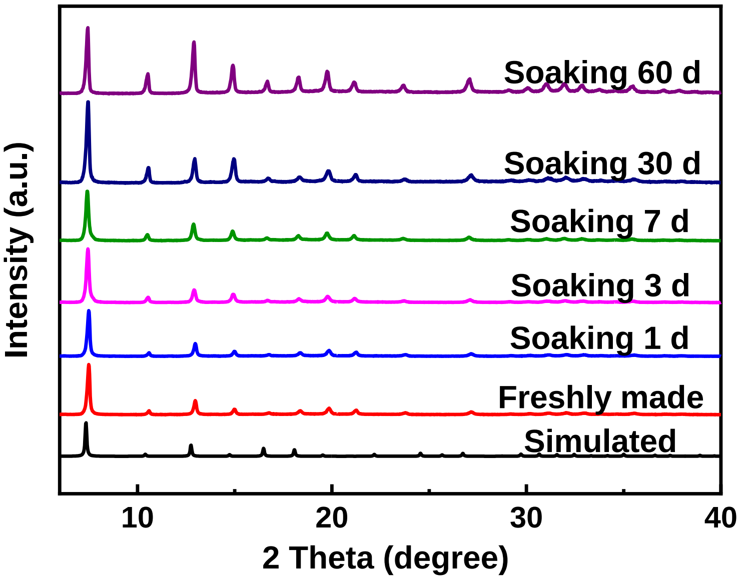

Figure S7. PXRD of ZIF-94 soaked in water for 1, 3, 7, 30 days, and 60 days.

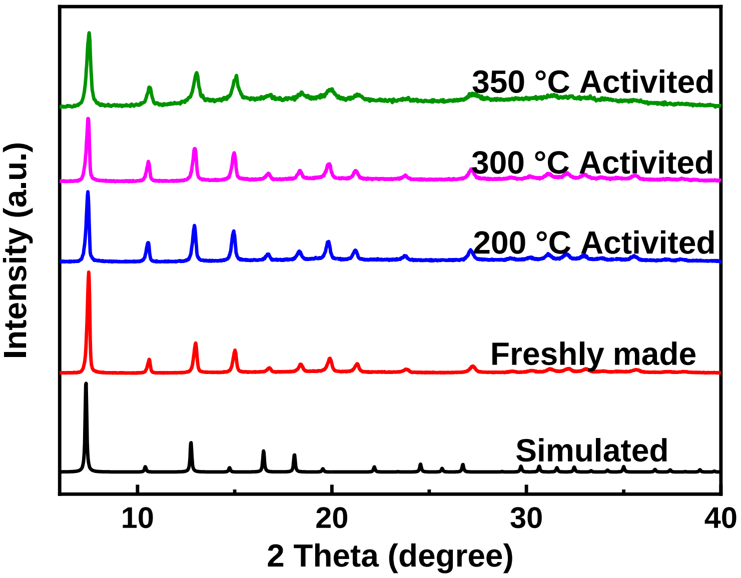

Figure S8. PXRD of ZIF-94 activated at different temperatures.

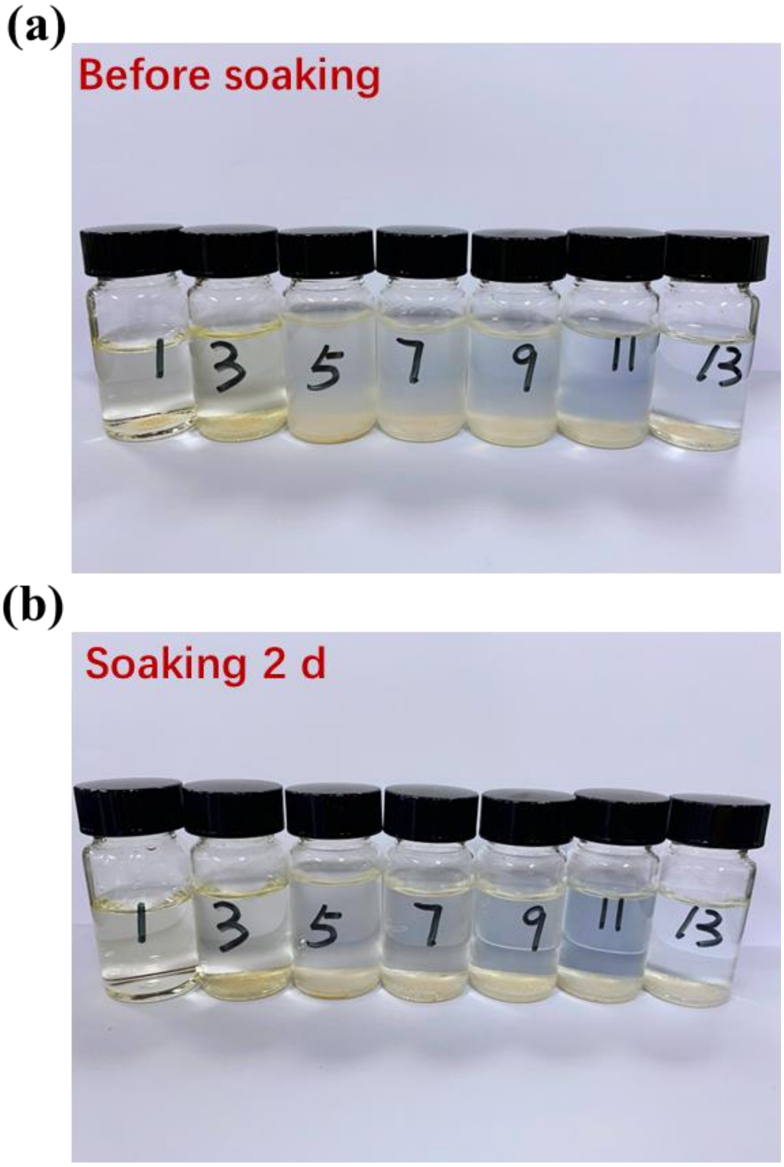

Figure S9. Images of ZIF-94 before and after soaking in different pH solutions. (**a**) before soaking, (**b**) soaking 2 days.

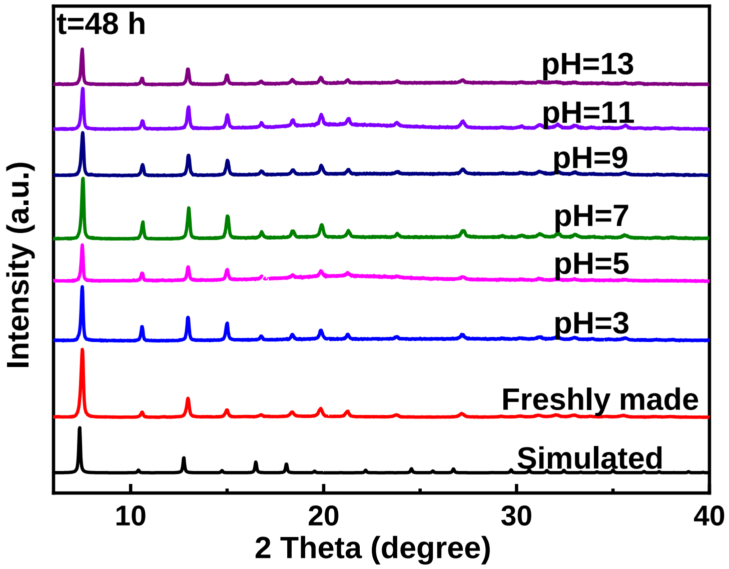

Figure S10. PXRD of ZIF-94 soaked in solutions with different PH values.

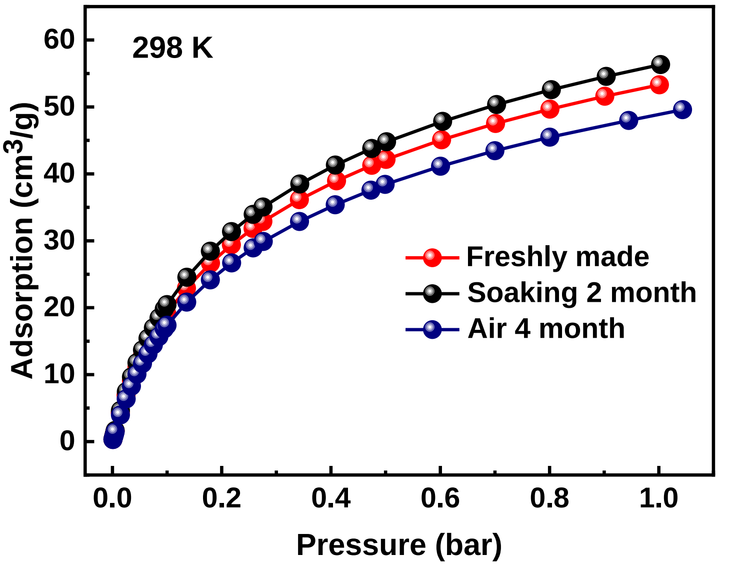

Figure S11. The adsorption isotherms of CO_2 under different conditions.

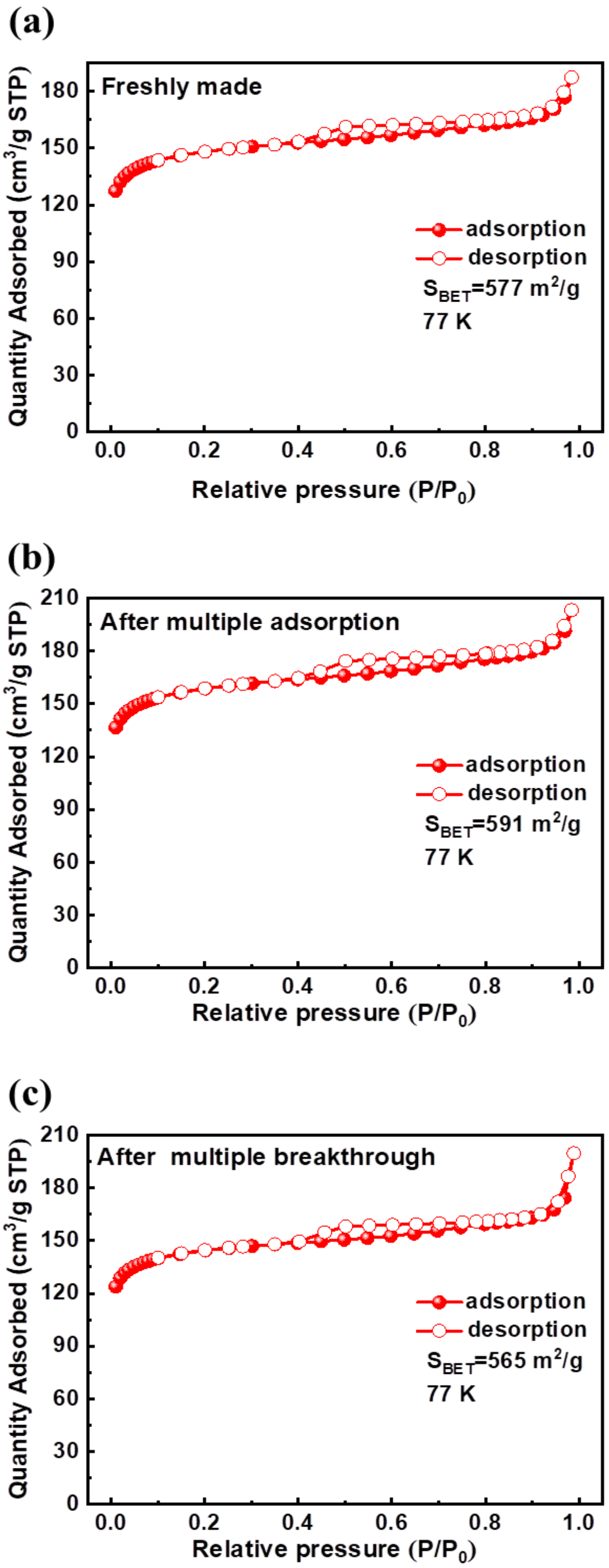

Figure S12. The N_2 adsorption isotherm at 77 K of the samples. (**a**) Freshly made, (**b**) Multiple adsorption experiments, (**c**) Dynamic breakthrough experiments.

Table S1. Dual-Langmuir-Freundlich fitting parameters for CO_2 and N_2 in ZIF-94 at 298 K.

	Site A			Site B		
	$q_{A,sat}$ cm^3 g^{-1}	b_A bar^{-1}	ν_A dimensionless	$q_{B,sat}$ cm^3 g^{-1}	b_B bar^{-1}	ν_B dimensionless
CO_2	56.24	0.95	0.95	28.64	9.28	1.00
N_2	0.42	24.99	1.36	24.3	0.33	1.13

Table S2. Dual-Langmuir-Freundlich fitting parameters for CO_2 and N_2 in ZIF-94 at 273 K.

	Site A			Site B		
	$q_{A,sat}$ cm^3 g^{-1}	b_A bar^{-1}	ν_A dimensionless	$q_{B,sat}$ cm^3 g^{-1}	b_B bar^{-1}	ν_B dimensionless
CO_2	40.89	15.47	0.94	50.11	1.20	0.99
N_2	1.04	8.22	1.17	41.72	0.31	1.07

Table S3. Comparation of CO_2 adsorption uptakes and selectivity for CO_2/N_2 around the top-performing ZIF.

ZIF	CO_2 adsorption (cm^3/g)	CO_2/N_2 Selectivity	Conditions (T, P)	Ref
ZIF-78	49.03 [a]	50.1 [c]	298 K,1 atm	[41]
ZIF-79	33.25 [a]	23.2 [c]	298 K,1 atm	[41]
ZIF-81	36.13 [a]	23.8 [c]	298 K,1 atm	[41]
ZIF-82	50.48 [a]	35.3 [c]	298 K,1 atm	[41]
ZIF-68	36.54 [a]	18.7 [c]	298 K,1 atm	[41,62]
ZIF-69	36.48 [a]	19.9 [c]	298 K,1 atm	[41,62]
ZIF-70	28.47 [a]	17.3 [c]	298 K,1 atm	[41,62]
ZIF-68	32.48 [b]	13.39 (15:85)	298 K, 0.10 MPa	[63]
ZIF-69	35.39 [b]	22.89 (15:85)	298 K, 0.10 MPa	[63]
ZIF-2	12.12 [a]	4.4 [d]	298 K, 1 bar	[48,64]
ZIF-4	17.83 [a]	8.2 [d]	298 K, 1 bar	[48, 64]
ZIF-5	8.98 [a]	16.3 [d]	298 K, 1 bar	[48, 64]
ZIF-8	8.68 [a]	3.6 [d]	298 K, 1 bar	[48, 64]
ZIF-9	36.83 [a]	14.8 [d]	298 K, 1 bar	[48, 64]
ZIF-95	19.26 [b]	18±1.7 [c]	298 K, 1 bar	[42]
ZIF-100	21.28 [b]	25±2.4 [c]	298 K, 1 bar	[42]
ZIF-8	15.64	7.22	298 K, 1000 mbar	[50]
ZIF-3	38.62	9.32 (50:50) [e]	298 K, 1 bar	[65]
ZIF-62	11.11	136.31	293 K,100 KPa	[66]
ZIF-67	14.23	5.56	298 K, 0.10 MPa	[67]
**ZIF-94**	**53.30**	**54.12 (15:85)**<br>**95.29 (50:50)**	**298 K, 1 bar**	**this work**

unit conversion: a. from $cm^3·cm^{-3}$ (literature reported) to $cm^3·g^{-1}$, b. from $mmol·g^{-1}$ (literature reported) to $cm^3·g^{-1}$, c. Henry's Law selectivity, d. Zero-pressure adsorption selectivity, e. GCMC simulations for CO_2 selectivity.

Table S4. Some typical materials with high CO_2 adsorption capacity.

Materials	CO_2 adsorption (cm^3/g)	CO_2/N_2 Selectivity	Conditions (T, P)	Ref
Cu-BTC	108.19 [a]	51.65	298 K, 1 bar	[68]
$Gly_{0.3}$@Cu-BTC	120.96 [a]	59.38	298 K, 1 bar	[68]
MIL-53(Al)	43.2	9.86 (15:85)	298 K, 1 bar	[69]
MIL-101(Cr)	53.31 [a]	21.23 (15:85)	298 K, 100 KPa	[70]
UiO-66	40.10 [a]	19.4	298 K, 1 bar	[71]
Fe_2(dobdc)	144.5	83.51	298 K, 1 bar	[72]
$Fe_2(O_2)$(dobdc)	98.1	45.49	298 K, 1 bar	[72]
Ni-MOF-74	123.2 [a]	32 (15:85)	298 K, 1 bar	[73]
Zn-MOF-74	122.08 [a]	NA [b]	298 K, 1 bar	[41]
Co-MOF-74	155.90 [a]	NA [b]	298 K, 1 bar	[41]
Mg-MOF-74	166.21 [a]	NA [b]	298 K, 1 bar	[74]
UTSA-16	93.63	NA [b]	298 K, 1 bar	[75]
CALF-20	91.17 [a]	230(10:90)	293 K, 1.2 bar	[76]
OM-CNS	67.42 [a]	14 (15:85)	298 K, 1 bar	[77]
FC4	64.29 [a]	14.2 (15:85)	298 K, 1 bar	[78]
NPC-1–600	112 [a]	27 (15:85)	298 K, 1 bar	[79]
BPL AC	45.70	26.43	298 K, 1 bar	[80]
Zeolite-13X	38.53 [a]	100.01	298 K, 1 bar	[80]
Silicalite-1	38.43	16.3	298 K, 1 bar	[81]

unit conversion: a. from $mmol \cdot g^{-1}$ (literature reported), b. Not available.

Reference

13. Banerjee, R.; Phan, A.; Wang, B.; Knobler, C.; Furukawa, H.; O'Keeffe, M.; Yaghi, O. M., High-throughput synthesis of zeolitic imidazolate frameworks and application to CO_2 capture. *Science* **2008,** 319, (5865), 939–943.
62. Liu, B.; Smit, B., Molecular simulation studies of separation of CO_2/N_2, CO_2/CH_4, and CH_4/N_2 by ZIFs. *The Journal of Physical Chemistry C* **2010,** 114, (18), 8515–8522.
63. Battisti, A.; Taioli, S.; Garberoglio, G., Zeolitic imidazolate frameworks for separation of binary mixtures of CO_2, CH_4, N_2 and H_2: A computer simulation investigation. *Microporous and Mesoporous Materials* **2011,** 143, (1), 46–53.
64. Thomas, A.; Prakash, M., Ionic liquid incorporation in zeolitic imidazolate framework-3 for improved CO_2 separation: A computational approach. *Applied Surface Science* **2021,** 562, 150173.
65. Wang, Y.; Jin, H.; Ma, Q.; Mo, K.; Mao, H.; Feldhoff, A.; Cao, X.; Li, Y.; Pan, F.; Jiang, Z., A MOF Glass Membrane for Gas Separation. *Angewandte Chemie International Edition* **2020,** 59, (11), 4365–4369.
66. Han, J.; Bai, L.; Jiang, H.; Zeng, S.; Yang, B.; Bai, Y.; Zhang, X., Task-specific ionic liquids tuning ZIF-67/PIM-1 mixed matrix membranes for efficient CO_2 separation. *Industrial & Engineering Chemistry Research* **2020,** 60, (1), 593–603.
67. Wu, Y.; Lv, Z.; Zhou, X.; Peng, J.; Tang, Y.; Li, Z., Tuning secondary building unit of Cu-BTC to simultaneously enhance its CO_2 selective adsorption and stability under moisture. *Chemical Engineering Journal* **2019,** 355, 815–821.
68. Kulak, H.; Polat, H. M.; Kavak, S.; Keskin, S.; Uzun, A., Improving CO_2 Separation Performance of MIL-53 (Al) by Incorporating 1-n-Butyl-3-Methylimidazolium Methyl Sulfate. *Energy Technology* **2019,** *7,* (7), 1900157.
69. Zhou, Z.; Mei, L.; Ma, C.; Xu, F.; Xiao, J.; Xia, Q.; Li, Z., A novel bimetallic MIL-101 (Cr, Mg) with high CO_2 adsorption capacity and CO_2/N_2 selectivity. *Chemical Engineering Science* **2016,** 147, 109–117.
70. Hu, Z.; Zhang, K.; Zhang, M.; Guo, Z.; Jiang, J.; Zhao, D., A Combinatorial Approach towards Water-Stable Metal–Organic Frameworks for Highly Efficient Carbon Dioxide Separation. *ChemSusChem* **2014,** 7, (10), 2791–2795.
71. Lou, W.; Yang, J.; Li, L.; Li, J., Adsorption and separation of CO_2 on Fe (II)-MOF-74: Effect of the open metal coordination site. *Journal of Solid State Chemistry* **2014,** 213, 224–228.
72. Chen, C.; Feng, X.; Zhu, Q.; Dong, R.; Yang, R.; Cheng, Y.; He, C., Microwave-assisted rapid synthesis of well-shaped MOF-74 (Ni) for CO_2 efficient capture. *Inorganic Chemistry* **2019,** 58, (4), 2717–2728.
73. Bae, T. H.; Long, J. R., CO_2/N_2 Separations with Mixed-Matrix Membranes Containing Mg_2(dobdc) Nanocrystals. *Energy & Environmental Science* **2013,** 6, (12), 3565– 3569.
74. Masala, A.; Vitillo, J. G.; Bonino, F.; Manzoli, M.; Grande, C. A.; Bordiga, S., New insights into UTSA-16. *Physical Chemistry Chemical Physics* **2016,** *18,* (1), 220–227.
75. Lin, J. B.; Nguyen, T. T.; Vaidhyanathan, R.; Burner, J.; Taylor, J. M.; Durekova, H.; Shimizu, G. K., A scalable metal-organic framework as a durable physisorbent for carbon dioxide capture. *Science* **2021,** *374,* (6574), 1464–1469.
76. Chen, C.; Yu, Y.; He, C.; Wang, L.; Huang, H.; Albilali, R.; Hao, Z., Efficient capture of CO_2 over ordered micro-mesoporous hybrid carbon nanosphere. *Applied Surface Science* **2018,** 439, 113–121.
77. Chen, C.; Huang, H.; Yu, Y.; Shi, J.; He, C.; Albilali, R.; Pan, H., Template-free synthesis of hierarchical porous carbon with controlled morphology for CO_2 efficient capture. *Chemical Engineering Journal* **2018,** 353, 584–594.
78. Kou, J.; Sun, L. B., Fabrication of nitrogen-doped porous carbons for highly efficient CO_2 capture: rational choice of a polymer precursor. *Journal of Materials Chemistry A* **2016,** 4, (44), 17299–17307.
79. McEwen, J.; Hayman, J. D.; Yazaydin, A. O., A comparative study of CO_2, CH_4 and N_2 adsorption in ZIF-8, Zeolite-13X and BPL activated carbon. *Chemical Physics* **2013**, 412, 72–76.
80. Wang, C.; Liu, J.; Yang, J.; Li, J., A crystal seeds-assisted synthesis of microporous and mesoporous silicalite-1 and their $CO_2/N_2/CH_4/C_2H_6$ adsorption properties. *Microporous and Mesoporous Materials* **2017**, 242, 231–237.
